# Immaterial animals and financialized forests: Asset manager capitalism, ESG integration and the politics of livestock

**DOI:** 10.1177/0308518X221121132

**Published:** 2022-08-18

**Authors:** Jeremy Brice, George Cusworth, Jamie Lorimer, Tara Garnett

**Affiliations:** Sustainable Consumption Institute and Manchester Institute of Innovation Research, Alliance Manchester Business School, 5292 University of Manchester, UK; Oxford Martin School, 6396 University of Oxford, UK; Hertford College, University of Oxford, UK; Oxford Martin School, 6396 University of Oxford, UK

**Keywords:** Universal ownership, ESG investment, food, finance, animal welfare, deforestation

## Abstract

This article uses interviews with responsible investment professionals to examine the extent to which institutional equity investors, and specifically ‘universal owners’ with highly diversified shareholdings, engage with public issues associated with livestock agriculture. As share ownership becomes increasingly concentrated, and the market for Environmental, Social and Governance investment products grows, these investors are increasingly involved in governing the activities of publicly traded corporations (including leading agribusinesses). This paper brings together political economy and marketization studies research to explore how universal owners become concerned about particular environmental and ethical problems, why they overlook other public concerns, and in what ways their selective engagement with ethico-political issues might be altering the content of food politics. Comparing universal owners’ engagements with farm animal welfare issues and with tropical deforestation within animal feed supply chains, we argue that these institutions engage with tropical deforestation because it presents a financially material risk to firms across multiple industries. By contrast, the specificity of farm animal welfare issues to agribusinesses means that they do not pose a material risk to the overall performance of universal owners’ highly diversified asset portfolios. Efforts to concern universal owners about livestock agriculture's social, environmental and health impacts thus generate a food politics which focuses primarily on risks to global economic systems and renders animals themselves distinctly immaterial.

Whether pledging to decarbonize their investment portfolios (Tett et al., 2021) or urging the Brazilian government to halt deforestation in the Amazon (Harris and Stott, 2021), institutional investors increasingly concern themselves with public issues. While such investor interventions may prove commercially advantageous, with some commentators estimating that $596bn was invested into sustainable investment products in 2021 (Kishan, 2022), there is reason to think they may also prove politically and environmentally consequential. Financial markets’ turn to sustainable and responsible investment has accompanied the emergence of a small group of asset managers and pension funds, sometimes termed ‘New Permanent Universal Owners’, which hold large ownership stakes in companies spanning entire stock markets. These organizations’ extensive shareholdings are often considered to grant them considerable influence over publicly traded corporations, enabling their investment decisions (in theory at least) to catalyze and channel global economic and ecological change (Crona et al., 2021; Galaz et al., 2018). Political economists therefore increasingly diagnose a new regime of corporate ownership and governance called ‘asset manager capitalism’ (Braun, 2021; Fichtner and Heemskerk, 2020), in which ‘ [i]nstitutional investors to a substantial degree establish the (…) conditions of possibility, on which capitalism in general and the capitalist production of nature in particular develops’ (Christophers, 2019: 755).

An emerging body of scholarship, sometimes termed ‘Universal Ownership Theory’, proposes that under asset manager capitalism these institutional investors will assume an increasingly prominent role in governing global ecological and social change. Its adherents argue that universal owners’ asset holdings now encompass so broad a swathe of the global economy that the costs of the uncompensated environmental, social and health harms created by their investee companies are increasingly being borne by other assets within their portfolios. They, therefore, predict that universal owners will pressure their investee companies to curtail these ‘negative externalities’ because doing so is in their financial interests (Jahnke, 2019b). However, universal owners often appear reluctant to assume this quasi-regulatory role by, for instance, supporting shareholder resolutions requesting that their investee companies address environmental and social issues (Fichtner et al., 2017). Meanwhile, their efforts to persuade investee companies to address issues such as climate change are increasingly being contested as a ‘politicization’ of financial markets which conflicts with their responsibility to maximize financial returns to their clients (Jahnke, 2019a). These developments call into question universal ownership theory's supposition that shifting patterns of asset ownership will directly lead financial institutions to concern themselves with environmental and social issues.

This paper responds to these challenges, and contributes to geographers’ theoretical understanding of institutional investors’ role in corporate governance under asset manager capitalism, through employing a marketization studies approach to explore how and why universal owners come to perceive environmental and social issues as costly economic externalities with which they might legitimately concern themselves. Mobilizing marketization studies scholarship's close attention to the calculative technologies and practices that inform which concerns are taken into account within (or excluded from) market transactions, we examine how these arrangements have mediated the absorption of the environmental and ethical issues surrounding livestock agriculture into financial markets. Over the past decade, several investor networks have emerged that support financial institutions in engaging with their investees to address Environmental, Social and Governance (ESG) issues associated with livestock production. These include the Global Investor Collaboration on Farm Animal Welfare (GICFAW), the Farm Animal Investment Risk and Return (FAIRR) Initiative and Investor Action on Antimicrobial Resistance. Meanwhile, the sustainability of livestock agriculture has become a matter of growing concern to the publics and policymakers for reasons including its high carbon and water intensity and its substantial role in deforestation and land conversion – whether through direct conversion of forests to pasture or the production of animal feedstuffs on deforested land (Cusworth et al., 2022; Galaz et al., 2018; Henders et al., 2015). As these growing environmental concerns intersect with longstanding unease over agricultural intensification's effects on animal welfare and anxieties about the implications of agricultural antibiotic use for human health, the socioecological impacts of livestock production are becoming increasingly controversial (Maye et al., 2021; Morris, 2018).

Despite livestock agriculture's environmental, economic and ethical salience – and in contrast to geographers’ extensive examination of financial institutions’ relationships with the environmental concerns surrounding fossil fuel production (Christophers, 2019; Langley et al., 2021) – institutional investors’ engagements with livestock-related public issues remain little explored. Although van Veelen (2021) has examined sustainable investment practitioners’ handling of greenhouse gas emissions from ruminant animals, climate change is only one of many issues which animate both public debates over the place of livestock within agri-food systems and the investor networks mentioned above (Maye et al., 2021; Morris, 2018). During 2021, these initiatives coordinated collaborative shareholder engagements focusing on issues from animal welfare (Amos et al., 2021) to tropical deforestation within meat supply chains (Ceres and FAIRR, 2021) and labour conditions within meat processing facilities (FAIRR, 2021). This paper argues that examining investor engagement with livestock issues complements existing scholarship on sustainable and responsible investment precisely because animal agriculture draws together many heterogeneous public issues. It thus opens up opportunities to investigate *which* environmental and ethical problems universal owners engage with and *why* they concern themselves with (or ignore) different issues.

Through comparing universal owners’ engagements with two such meat and livestock issues – farm animal welfare and tropical deforestation – this paper addresses three questions pertaining to the shifting relationship between finance and politics under asset manager capitalism. First, why have universal owners begun to concern themselves with environmental, social and health issues to which they formerly appeared indifferent? Second, why do they concern themselves more readily with some such issues than others? Third, in what ways might the content of food politics (and politics generally) be changing as its constituent issues are selectively absorbed (or ‘internalized’) into financial markets? In so doing we aim to understand how efforts to enrol financial institutions into the negotiation of ethico-political issues might be reshaping both the contours of financial markets and the content of political debate under asset manager capitalism.

The following two sections introduce existing research examining financial institutions’ engagements with environmental and ethical issues. The second section provides a detailed introduction to universal ownership theory, arguing that this body of scholarship has yet to explain how and why ethico-political issues become financial risks that might concern institutional investors. In response, the third section draws upon marketization studies research to argue that this process is best understood through examining how changing configurations of calculative devices and practices mediate the internalization of ethico-political concerns into financial markets. A brief methodology section then outlines how this paper draws on interviews with responsible investment professionals to explore universal owners’ engagements with the issues of farm animal welfare and of tropical deforestation. Next, the paper's analysis addresses our first question by examining universal owners’ increasingly active engagement with tropical deforestation within meat and animal feed supply chains – arguing that this issue's repositioning as a contributor to climate change has transformed it into a systemic risk to global economic growth. The paper then addresses our second question by examining universal owners’ engagements with farm animal welfare, suggesting that they have neglected this issue because its specificity to the agribusiness sector prevents it from materializing as a systemic risk to universal owners’ investment portfolios. The conclusion responds to our final question, arguing that universal owners’ engagements with livestock issues generate a food politics focused on addressing systemic risks to the profitability of global economic systems – one which renders animals themselves immaterial.

## Universal ownership: Politicizing finance?

Many accounts link the emergence of asset manager capitalism, and of the New Permanent Universal Owners, to the investment of a growing proportion of capital in index funds (Fichtner et al., 2017). These products do not seek to select assets that will yield returns greater than the market average, but simply to replicate the composition and financial performance of a benchmark stock index such as the S&P 500. Their popularity has grown rapidly since the 2008 financial crisis due to their capacity to produce average returns comparable to those of actively managed funds at considerably lower costs (Braun, 2016, 2021), enabling a few index fund providers to accumulate extensive shareholdings in companies spanning entire stock markets (Jahnke, 2019b). By 2018, the three largest such firms – US asset managers BlackRock, Vanguard and State Street – held an average combined ownership stake in S&P 500 companies of over 20% (Fichtner and Heemskerk, 2020). By 2016, the USA's five largest asset managers had also accumulated combined shareholdings of 10–30% in many major agribusiness corporations (Clapp, 2019).

Some scholars attach considerable significance to the emergence of universal owners whose: ‘cumulative long-term return is determined not merely by the performance of each individual firm it owns, but by the performance of the economy as a whole’ (Hawley and Williams, 2000: xv). By these accounts, the expansion of such institutions’ asset portfolios to encompass much of the economy means that if the activities of a firm in which a universal owner is invested cause uncompensated harms to others then the costs of these negative externalities will likely be borne by *other assets within their portfolio* (Jahnke, 2019b). For instance, using antibiotics as livestock growth promoters may make an agribusiness firm highly profitable. However, the costs of antibiotic-resistant infections to a universal owner's other assets are likely to equal or exceed the returns that it derives from its shareholding in this company. Such accounts suggest that it is difficult for universal owners to profit from activities that create negative externalities and that their financial interests lie in persuading investees to curtail their production (Braun, 2021; Fichtner and Heemskerk, 2020).

Because index fund providers do not actively decide which assets to purchase, they are considered likely to ‘internalize’ negative externalities through engagement with investee companies rather than through changes in asset pricing or capital allocation. Such firms’ considerable combined shareholdings in most publicly traded corporations arguably enable them to exercise both sufficient voting power and sufficient coordination to exert significant pressure upon investee companies (Jahnke, 2019b). As the legal owners of assets, which they administer on their clients’ behalf, asset managers may propose and vote on resolutions presented at the Annual General Meetings (AGMs) of companies in which they own shares. This enables them to request that company management address specific environmental and social issues or demonstrate their disapproval of company policy through blocking executive remuneration packages and the reappointment of recalcitrant board members – if a large enough proportion of shareholders can be convinced to support or reject key resolutions. Universal owners’ extensive equity portfolios have made their backing increasingly instrumental in determining the success or failure of shareholder resolutions, lending weight to their private engagements with investee companies (Fichtner and Heemskerk, 2020). Although universal owners are in practice often reluctant to support shareholder resolutions on ESG issues (Fichtner et al., 2017), Jahnke (2019a: 17) argues that they nevertheless ‘have the opportunity to act as stewards of their portfolio companies, sensitizing them to changes in public attitudes’.

However, institutional investors’ attempts to align investee companies’ business practices with (their perception of) wider publics’ interests and values are increasingly contested. Collaborative investor engagements on climate change and on gun control in the United States have encountered resistance from industry groups arguing that such initiatives conflict with their clients’ financial interests and thus constitute ‘political’ interference in the operation of markets (Jahnke, 2019a), as has Australian banks’ adoption of responsible lending policies prohibiting investment in companies which export living animals (McKillop, 2021). Claims that asset managers’ political convictions might be determining their investment decisions are particularly damaging for institutional investors (including universal owners) which are subject to fiduciary duty legislation obliging them to invest in a fashion which prioritizes their clients’ interests (Clapp, 2019). Although recent reforms have clarified that investment fiduciaries should consider the financial risks posed by environmental and social issues as part of their duty to invest prudently (Sullivan et al., 2015), institutional investors still interpret fiduciary duty primarily as a responsibility to maximize financial returns to asset owners without incurring excessive exposure to risk (Christophers, 2019; Ouma, 2020). Fiduciary duty requirements are therefore often considered to oblige institutional investors to exclude purely ethico-political concerns from consideration when making investment decisions (Kish and Fairbairn, 2018; Leins, 2020).

Such attempts to purify financial markets of ethical considerations seemingly clash with expectations that the maturation of universal ownership will propel the internalization of environmental and social externalities hitherto considered political in character. While existing research on universal ownership acknowledges that shifts in the location of this boundary between ‘the political’ and ‘the economic’ engender controversy and contestation (Jahnke, 2019a), it rarely investigates *why* issues hitherto classified as being ethico-political might be repositioned as matters of economic concern to financial institutions. It, therefore, struggles to explain *how* financial institutions might justify engaging with such concerns and *why* the location of the border separating financial markets from political issues might change over time. To gain greater purchase upon these processes the following section turns to marketization studies scholarship, which traces the processes through which market devices both generate new political issues and re-internalize public concerns.

## Marketization studies: Economizing politics

While financial professionals may strive to disentangle their investment decisions from ethico-political considerations, geographers examining the constitution of markets (or processes of marketization) suggest that this task may be more difficult than it appears. Such researchers argue that market arrangements facilitate calculative, utility-maximizing decision making through orienting buyers’ and sellers’ attention towards characteristics of goods and services deemed salient to the transaction at hand while excluding other aspects of these items from consideration (Barry, 2002; Geiger et al., 2014). Financial analysts’ capacities to evaluate assets purely in terms of their ‘fundamental’ characteristics of risk and expected return (Leins, 2020) thus depend upon numerous ‘framing’ operations which establish ‘a boundary between relationships and events which are internalized and included in a decision or, by contrast externalized and excluded from it’ (Callon, 1998: 15).

These unconsidered and unvalued externalities often generate new political issues through spurring those affected by them to form new publics which demand that market arrangements be revised to take their interests into account (Barry, 2002). For instance, while the impacts of water pollution on people and wildlife living downstream of intensive poultry units (IPUs) are rarely considered by those who price either poultry products or shares in poultry-producing companies, they may provoke vociferous local mobilizations demanding the remediation (or internalization) of IPU effluent (Caffyn, 2021). Callon (2009) therefore characterizes a well-functioning market as one sufficiently attentive to those who articulate the harms caused by its externalities that it revises its framing processes to accommodate their concerns (Kish and Fairbairn, 2018). By this account markets, far from being spaces set apart from normative concerns, are prominent sites of controversy where passionate debates rage over which issues should be considered during economic valuation and exchange (Geiger et al., 2014).

Financial markets are no exception, as is demonstrated by numerous efforts to measure the environmental and social impacts generated by publicly traded companies and by the development of financial devices (from green bonds to social impact funds) designed to channel capital towards more ‘ethical’ or ‘sustainable’ assets (Barman, 2015; van Veelen, 2021). These innovations aim to reshape actors’ understandings of which public issues should be considered (or not) within financial transactions through re-tracing the market frames within which valuation occurs. Yet internalizing and marketizing new concerns entails considerable investment in new sociotechnical apparatuses of measurement, comparison, (e)valuation and framing, from auditing protocols to company sustainability ratings. This transforms the invention and imposition of such devices into a central focus of political contestation (Arjaliès and Bansal, 2018; Callon, 2009).

These points can be illustrated by examining recent efforts to refashion the calculative rationalities of financial markets by introducing technologies of ESG integration. The term ESG investing was formalized in 2006 through the publication of the UN Principles for Responsible Investment (UNPRI), whose signatory financial institutions committed to ‘incorporate Environmental, Social and Governance (ESG) issues into investment analysis and decision-making’ (UNPRI, n.d.). Financial institutions which commit to this process of ‘ESG integration’ must identify and consider public issues which might subsequently impact asset prices or revenues when valuing assets and deciding where to invest their capital. ESG integration, therefore, attempts to transform environmental and social issues into economic problems amenable to consideration within the framework of financial analysis by evaluating them in terms of their ‘financial materiality’ – their capacity to make a difference to the future value of assets (Leins, 2020; Parfitt, 2020).

Parfitt (2020) suggests that this economization of environmental and social issues has both legitimized ESG integration within mainstream financial institutions and rendered it compatible with fiduciary duty regulations. ESG integration promises institutional investors a means of distinguishing public issues which may affect returns on their clients’ investments (and therefore present ‘economic’ problems) from purely normative concerns whose consideration might contaminate financial calculation with a ‘political agenda’ (Christophers, 2019; Jahnke, 2019a; Ouma, 2020). This has facilitated its rapid adoption among institutional investors (Leins, 2020), with some sources estimating that by 2020 over $35tn of assets – approximately 36% of total Assets Under Management (AUM) across major developed markets – were being managed under ESG principles (Kishan, 2022). However, this stricture that environmental and social concerns should be considered only if they are financially material reinscribes the border between the economic and the ethico-political in ways which limit the range of issues that ESG investors may address. While ESG integration enlarges the framework of financial analysis to accommodate environmental and social concerns that present risks to the future financial performance of assets, it reaffirms the externalization of financially *im*material issues.

These arguments hold two important implications. First, they suggest that institutional investors’ understandings of which concerns should inform investment decisions and shareholder engagements may shift over time as the calculative devices and practices employed within financial markets change. As such, it is insufficient to make inferences about financial institutions’ attitudes towards the internalization of externalities based upon changing patterns of equity ownership alone, as do many existing works on universal ownership (e.g. Braun, 2021; Fichtner and Heemskerk, 2020). Second, they hint that universal ownership theory's contention that the advent of asset manager capitalism will promote the internalization of externalities into financial markets may operate at too high a level of generality. Marketization studies scholarship highlights that public issues are internalized into markets only through intense struggle among the specific coalitions of actors which assemble around them. Financial markets’ adoption of calculative norms and equipment which internalize one hitherto-overlooked issue (e.g. greenhouse gas emissions from ruminant livestock) may thus be entirely compatible with the continued neglect of another (say, animal welfare) (Callon, 2009; Geiger et al., 2014).

Marketization studies scholarship thus foregrounds a different set of questions about financial markets’ engagement with public issues. While universal ownership theory asks *whether* index fund providers are predisposed to internalize the environmental and social harms caused by their investees, marketization scholarship instead begins by exploring *how* market arrangements are refashioned to accommodate specific public issues within the frame of financial calculation. This attention to the innovations and contestations which transform particular concerns from ethico-political issues to be disputed into risks subject to financial calculation offers a means of disaggregating the great internalization of externalities posited by universal ownership theory. It thus raises questions about *why* financial markets – and universal owners specifically – might internalize some public issues while neglecting other pressing concerns. Posing this question subtly changes the stakes of scholarly engagement with the phenomenon of universal ownership. It invites researchers to investigate how the advent of universal ownership might be *redistributing* attention and neglect within financial markets (and thus reshaping patterns of internalization and externalization), and to consider what implications this transformation might hold for those affected by the activities of universal owners’ investee companies.

## Methodology

This paper mobilizes the capacity of debates over livestock agriculture to bind together numerous environmental, ethico-political and health issues to address these latter questions (Maye et al., 2021; Morris, 2018). The following sections draw on 50 interviews conducted during 2021 over Zoom or Microsoft Teams with representatives of organizations involved in efforts to establish meat and livestock issues as matters of concern to institutional investors (including NGOs, responsible investment networks, ESG research firms and financial institutions participating in relevant responsible investment initiatives). Interviewees included employees of 10 asset management firms headquartered in the UK, the United States and the EU, whose AUM varied from approximately £1.5bn to over £1tn. These firms ranged from specialist asset managers providing actively managed ethical investment products to retail or charitable investors to large international firms primarily serving institutional investors such as pension funds (some of which maintained extensive holdings in index funds). This enabled the project to examine which livestock-related environmental and ethical concerns were taken into account by financial institutions employing multiple responsible investment strategies and representing a diverse mixture of clients. These interviews were conducted on condition of anonymity and the names of all interviewees, and of their employers, have been replaced with pseudonyms.

Taking inspiration from marketization studies scholarship's injunction to study the devices and processes through which ethico-political concerns are internalized into market frames, the following sections compare institutional investors’ engagements with two livestock issues which are subject to elaborate infrastructures of measurement, comparison and evaluation. Our first issue, tropical deforestation, has risen rapidly up the ESG agenda since the signing in 2014 of the New York Declaration on Forests, which committed numerous consumer goods companies and financial institutions to eliminate deforestation within agricultural commodity (including beef and soy) supply chains by 2020. These commitments spurred the development of multiple scoring and benchmarking systems (including the WWF soy scorecard and environmental NGO Global Canopy's Forest 500 benchmark) which assess companies’ progress towards achieving these zero deforestation targets and thus their exposure to deforestation-related ESG risks (Thomson, 2020). They also produced the Investor Initiative for Sustainable Forests (IISF), an investor network that supports institutional investors in undertaking shareholder engagements aimed at eliminating deforestation within beef and soy supply chains.

Our second issue, farm animal welfare, is also a prominent site of experimentation with new techniques for translating ethico-political concerns into ESG risks. In 2012, farm animal welfare became the focus of the first dedicated ESG rating system for food businesses, the Business Benchmark on Farm Animal Welfare (BBFAW), and in 2014 the GICFAW – the first investor network focused on livestock issues – was launched (Amos et al., 2021; McLaren and Appleyard, 2022). Both issues have thus accreted dense networks of calculative devices designed to establish them as ESG risks which should concern institutional investors. In examining the extent to which universal owners engage with these two issues, the following sections explore *how* these influential investors become concerned about new issues and *why* they might concern themselves with some public issues while overlooking others.

## Financialized forests

“We made a commitment that we would start a hands-on engagement programme, targeted at some of the largest companies in our portfolios, on their strategic management of climate change (…) as responsible stewards of those assets, we have the responsibility to (…) ensure that they are managing these key strategic, or key long-term, risks that climate change poses. And because food was one of the sectors that we targeted, deforestation naturally became a big focus because, in our view, the food sector essentially has two levers to pull to try and decarbonize. One is obviously their product portfolios (…) But then the second significant lever is what's happening with their supply chains, and making sure that they’ve rooted out deforestation”

When Elise (an ESG analyst working for a large asset manager occupying a position of universal ownership) explained why tropical deforestation featured prominently within her employer's shareholder engagement programme, she immediately highlighted the greenhouse gas emissions generated when forested land is converted into pasture for cattle or farmland producing soy for use in animal feed. In this respect, her account exemplified a recent shift among both activists and investors from approaching tropical deforestation as an issue of intrinsic moral concern towards presenting it as one contributor to global climate change – in their view the greatest ESG risk of all (e.g. Ceres, 2020; Jones et al., 2020). This repositioning of tropical deforestation as being important due to its climate change implications has assisted efforts to establish it as a matter of concern to universal owners in several ways.

First, this move linked shareholder engagement on tropical deforestation closely to an ESG issue that institutional investors were already beginning to accept as being material to the future financial performance of assets across their portfolios. As Christophers (2019) notes, many mainstream institutional investors no longer consider climate change a primarily ethical or environmental issue and instead increasingly treat it as a financially material risk that must be accounted for within the economic models used to value assets. The reasons for this are complex. Long-term institutional investors’ concern over climate change is informed partly by economic modelling studies which project that the costs of climate change impacts will substantially reduce future global GDP growth. Actors such as the UNPRI (UNEP-FI and UNPRI, 2011) argue based on such research that climate change presents a ‘systemic’ or ‘unhedgeable’ risk to which universal owners’ highly diversified portfolios are particularly exposed because their performance is associated with overall economic growth (Hawley and Williams, 2000). However, it also reflects anxieties that impending policy changes may soon reduce the profitability and value of companies within emissions-intensive industries (Crona et al., 2021; van Veelen, 2021).

It is therefore significant that growing attention to tropical deforestation's contribution to climate change has situated the land conversion footprint of beef and soy production within a broader environmental issue which implicates multiple industries. Production of widely used commodities including palm oil, timber, paper and rubber is often identified as driving tropical deforestation (Henders et al., 2015), meaning that this ESG risk might plausibly affect companies from food producers to automobile manufacturers (Ceres, 2020). This appears to have been particularly important in convincing universal owners that future regulations targeting deforestation risk commodities might affect a sufficiently large group of their investee companies to impact materially on returns across their equity portfolios. Victoria, a shareholder engagement professional employed by a small asset manager specialising in ethical investment strategies, which was active in multiple anti-deforestation initiatives, argued that in consequence:

“we’ve been able to really build a movement in the finance and investor community of people who are concerned about [deforestation] (…) a lot of investors are – I mean, like BlackRock – expecting all of the companies they invest in to have net zero by 2050 plans developed. For companies with exposure to deforestation, that's going to play a big role in whether or not they meet those goals. So that has also helped drive the material risk.”

Much as universal ownership theory predicts, these considerations had prompted two universal owners to undertake sustained shareholder engagement programmes intended to reduce food businesses’ exposure to tropical deforestation and employ relatively confrontational engagement tactics against company managers who did not respond constructively. For the firm which employed Nadia, another shareholder engagement professional, this entailed voting for AGM resolutions requesting that unresponsive investee companies introduce stronger anti-deforestation policies and against the reappointment of their boards of directors:

Climate change and deforestation, for instance, are part of our voting approach. And the [benchmark] we use for deforestation is Forest 500. That benchmark, again, [we are] looking at the laggards, setting minimum expectations. And then, if we are not satisfied from the outcomes of engagement, we might recommend votes against (…) once we actually link up vote implications with poor ESG performance it becomes very clear to companies (…) that this is – it's business critical. Unpriced externalities can no longer be ignored.

Nadia's reference to the Forest 500 benchmark – which scores and ranks the quality of corporations’ anti-deforestation policies and targets, and of their reporting of progress towards achieving these targets (Thomson, 2020) – is significant. A low Forest 500 ranking suggests that a firm is more likely to purchase commodities grown on deforested land and thus at greater risk of costly reputational damage or regulatory action. Calculative devices such as Forest 500 are typically designed in the hope that financial institutions will use them to invest preferentially in high-scoring firms and to pressure low-scoring companies through divestment or engagement, thus incentivizing businesses to achieve high ethical and environmental standards (Mehrpouya and Samiolo, 2016). Nadia's firm employed the benchmark in precisely this manner – using it to assess which of their investees were most exposed to tropical deforestation, allocate their limited engagement resources to these companies and thus (they hoped) to reduce their portfolio's exposure to deforestation risk efficiently. They sought thereby to address deforestation in a manner that optimized the financial return provided to clients by their asset portfolio (Callon, 1998). Elise's firm had taken this calculated approach to investment stewardship further by adopting shareholder engagement procedures under which, if an investee company failed repeatedly to improve its management of deforestation risks, they would oppose the reappointment of its board and sell any shares held by their ESG funds:

We have to have clear evidence that you are really not meeting our expectations (…) and are not responding to investor pressure to improve. So, we won't say ‘Oh, you didn't – you don't have a deforestation policy. We’re divesting you.’ But we *will* say, ‘We have been asking you now for eighteen months to have a deforestation policy in place, and for it to cover X, Y and Z. And you have not indicated to us that that is imminent. Hence, we will be divesting and voting against.’

Recent events suggest that the prevalence of such deforestation-focused engagement, and even divestment, programmes may be growing among large asset managers. In 2020, seven European pension funds and asset managers threatened to divest from beef producers and commodity traders active in Brazil, and from Brazilian government bonds, if these organizations did not act to address deforestation in the Brazilian Amazon. Nordea Asset Management and Robeco Global Asset Management subsequently executed this threat by selling their holdings in Brazilian meatpacking conglomerate JBS (Harris and Stott, 2021), while in 2021, UK-headquartered asset manager Legal & General Investment Management divested from China Mengniu Dairy Corporation due to its lack of a zero deforestation policy (Amorim, 2021). Also in 2021, shareholders in commodity traders Bunge and Archer-Daniels-Midland (ADM), and in restaurant group Bloomin’ Brands, passed resolutions requesting that these companies introduce more stringent traceability programmes for deforestation risk commodities and publicly disclose the proportion of their products certified as deforestation-free (Dhanasarnsombat, 2021). As Victoria noted, it would be difficult to pass such resolutions without the support of universal owners due to the size of their combined shareholdings. The increasing success rate of deforestation-related shareholder resolutions, therefore, suggests a growing acceptance among such institutions that exposure to tropical deforestation poses a material risk to the future performance of their portfolios:

we had a proposal with [name of company] in the fall, and BlackRock voted in favour (…) so did State Street and Vanguard. And we’ve seen that trend continue (…) there has been a very notable shift in the last year or two with the mainstream asset managers (…) more established issues, deforestation being one of them at this point, we’ve seen just *massive* votes this past season on proposals related to those issues.

The prevalence of investor interventions to address deforestation within meat, dairy and animal feed supply chains – and especially of deforestation-related divestments from food businesses – should not be overstated (Harris and Stott, 2021). However, the growing number of successful shareholder resolutions on this issue does suggest that universal owners are becoming increasingly inclined to take tropical deforestation into account in their stewardship of investee companies and that these interventions may be securing modest changes in food businesses’ management of their supply chains. Notably, in response to the shareholder resolutions detailed above Bunge and ADM introduced new deforestation policies incorporating commitments to implement more stringent product traceability requirements, eliminate deforestation from their supply chains (by 2025 and 2030, respectively), and disclose their progress towards these goals (Green Century Capital Management, 2021). Universal owners’ newfound concern over the ESG risks posed by tropical deforestation may thus be persuading publicly traded food businesses to commit to taking limited steps towards internalizing this externality (Callon, 2009; Geiger et al., 2014). However, it is currently unclear, given the recency of these shareholder resolutions, whether investors will be able to enforce compliance with these anti-deforestation commitments and whether their growing concern over deforestation risk will devalue, or deter future investment into, companies exposed to it.

Examining universal owners’ engagements with tropical deforestation have nevertheless illustrated that convincing them to perceive this issue as a financially material risk, and to integrate it into their shareholder engagement programmes, is itself a significant achievement that has required considerable effort. Research linking climate change to slower global GDP growth, and the threat that potentially costly changes to anti-deforestation regulations might affect companies in multiple deforestation risk sectors, both contributed to demonstrating that tropical deforestation might affect overall returns to a universal owner's portfolio. Meanwhile, the creation of deforestation-focused benchmarks rendered different businesses’ exposure to regulatory action calculable and comparable, making tropical deforestation visible as a financial risk that was distributed unevenly across universal owners’ asset portfolios and could therefore be mitigated through targeted shareholder engagement and divestment. Tracing the internalization of tropical deforestation into the frame of financial calculation has thus illustrated that universal owners’ concern over this issue did not, as universal ownership theory suggests, arise directly or inevitably from their growing equity holdings in deforestation risk companies such as agribusinesses. Rather, the emergence of these new knowledges and calculative devices has helped to reframe universal owners’ understanding of their own economic interests by convincing them that tropical deforestation could reduce future financial returns across their portfolios. These market devices have thus mediated deforestation's transformation from an ethico-political issue into a systemic financial risk – rendering it legitimate and justifiable according to the calculative conventions of ESG investing for universal owners to concern themselves with deforestation and to pressure their investees to internalize this externality (Leins, 2020; Parfitt, 2020). The following section further develops this marketization studies-inflected analysis by exploring what role the calculative technologies of ESG investment have played in shaping universal owners’ engagement with – and limiting their concern over – animal welfare issues.

## Immaterial animals

While investors’ increasing engagement with tropical deforestation reflects relatively recent concerns about the economy-wide risks of climate change, animal welfare issues have long presented a prominent site of articulation between markets and morality. Nineteenth and early 20th centuries anti-cruelty movements sought to prohibit commercial practices deemed to cause morally unjustifiable suffering to animals, while during recent decades animal welfare has been economized through numerous product certification schemes which promise producers who exceed basic standards of animal husbandry a premium price for their products (Buller and Roe, 2018). Such market arrangements address animal welfare concerns less through proscribing controversial agricultural practices than through transforming animal welfare itself into a quality that is material to the value of animal products (Miele, 2011). Financial markets’ engagements with animal welfare have recently undergone a similar transformation. Since at least the 1990s certain UK-headquartered financial institutions have offered funds that exclude companies involved in animal testing from their portfolios, targeting clients who consider these activities morally objectionable (Sparkes, 2010). However, sustained efforts have been made to economize the issue of farm animal welfare since the launch in 2012 of BBFAW which – much like Forest 500 – assesses the quality of major food businesses’ animal welfare policies and of their public disclosures regarding animal welfare practices within their supply chains (Amos et al., 2021; McLaren and Appleyard, 2022). As Thomas – a responsible investment professional who had participated in developing BBFAW – recounted in an interview, this benchmark was designed explicitly to broaden the rationale for taking farm animal welfare into account within investment and shareholder engagement processes beyond purely ethical considerations:

we interviewed investors, we said, “What would it take to make [farm animal welfare] of relevance to you?” and they said, “Well it's not financially material. But a tool like a benchmark, which allows us to differentiate between companies on the basis of how they manage this as a new and emerging risk, would be very interesting to us.” So the early logic (…) is that while nobody really cares about animal welfare, they *do* care about how companies manage horizon risks, or risks that have yet to manifest themselves.

BBFAW's creators thus aimed to establish farm animal welfare as an issue about which financial institutions should be concerned not merely because it might conflict with their clients’ *moral* values but because strong animal welfare performance signals that a company is generally well managed and therefore less financially risky. Complementing this general claim, BBFAW's advocates also argued that agribusinesses that attain high animal welfare standards will deliver superior financial performance because they enjoy easier access to higher value market segments, reduced exposure to reputationally damaging animal welfare scandals and greater adaptability to new regulations (Sullivan et al. 2012). BBFAW's subsequent development, alongside previous research (McLaren and Appleyard, 2022), suggests that such attempts to persuade investors that poor management of farm animal welfare presents material ESG risks have enjoyed some success. By May 2021, GICFAW's annual collaborative shareholder engagement campaign had gained 39 members, representing a combined total AUM of £2.1tn and including large active asset managers such as Aberdeen Standard Investments, Aviva Investors and BMO Global Asset Management (Amos et al., 2021). Nevertheless, several interviewees argued that most mainstream asset managers had yet to recognize farm animal welfare issues as being financially material or integrate them meaningfully into their ESG risk management frameworks. Thomas attributed animal welfare's patchy materialization as an ESG risk partly to enduring difficulties in demonstrating a measurable relationship between a company's financial performance and the quality of its animal welfare disclosures, explaining that:

I think there was no understanding of the pathways whereby animal welfare would manifest itself, other than (…) in a scandal. You know, Marks & Spencer has calves on a farm who are treated badly, or left to go hungry, or whatever. So it was only if there was a scandal or a news story that investors would even look. And then they would react as investors would: what does that mean for reputation, cost, etcetera? (…) But does better welfare translate into better financial outcomes? It's moot, there's actually limited evidence.

Thomas thus argued that animal welfare's transformation into a financial risk had been impeded by a paucity of evidence linking animal welfare performance to the financial returns provided by individual *firms* (implying that more extensive corporate disclosure and research might overcome this obstacle). However, interviewees employed by large asset managers serving institutional investors rarely identified this evidentiary problem as a constraint. Instead, they suggested that farm animal welfare's very nature as an issue presented more fundamental obstacles to its materialization as an ESG risk within their firms, ones unlikely to be overcome simply through demonstrating a relationship between animal welfare and firm-level financial performance more persuasively. As Daniela, a shareholder engagement professional working for a financial institution with an AUM of over £1tn, explained:

ESG is very, very wide and there has been a focus on just a few issues. It's impossible to cover everything (…) climate change is systemic. Um, so we don't really have a choice, we must do something about that. (…) But it's also a combination of what kind of impact can we have and across how many sectors do we have that? (…) That is the kind of thought that goes into it when we set, um, set our strategy. Unfortunately, many times it will mean stepping away from areas that are really important as well.

It was perhaps significant that Daniela's firm held much of its clients’ capital in equity index funds. Daniela was quick to highlight that despite the breadth and scale of her employer's equity portfolio, it invested in only a relatively small number of companies within a single sector that raised farm animals. That only companies involved in livestock production are directly responsible for managing farm animal welfare is perhaps self-evident. However, it is also important because Daniela's employer, as a universal owner, viewed issues that only affected firms within a single sector as being unlikely to impact noticeably on the overall financial performance of its funds. Engagement professionals such as Daniela, therefore, tended to concentrate on ESG issues which they deemed either to present material risks to companies across multiple industries or to present a systemic risk to the entire economy (and thus to their employer's portfolio).

Daniela's position substantiates universal ownership theory's contention that universal owners evaluate their financial returns and define their economic interests at the level of their portfolio (and by extension the entire economy) rather than that of individual assets (Fichtner and Heemskerk, 2020; Hawley and Williams, 2000). However, it also challenges some of this body of scholarship's predictions, for it suggests that when this practice combines with the calculative technologies of ESG integration it *impedes* the internalization of farm animal welfare into such firms’ investment and shareholder engagement processes rather than encouraging it. Daniela considered that even if poor farm animal welfare management could be shown to present a significant financial risk to individual *investee companies,* its materiality to her firm's portfolio would nevertheless remain negligible. It would therefore be difficult to persuade her employer that animal welfare issues presented a material risk to their clients’ investments which her engagement programme should address. While Daniela acknowledged that sector-specific issues such as farm animal welfare might also be ‘really important’ in ethical terms, their immateriality to most of her employer's investee companies meant that in practice her firm remained distinctly *un*concerned about them.

Daniela's account thus illustrates how the calculative technologies of ESG integration not only mediate but also constrain the impulse to internalize environmental and social externalities which universal ownership theory identifies. In classifying sector-specific issues such as farm animal welfare as being immaterial to the financial performance of universal owners’ portfolios, ESG integration arrangements excuse these influential investors from taking these concerns into account within their investee engagement programmes. Mobilizing marketization studies’ close attention to the calculative norms and devices which underlie ESG integration thus leads us to temper universal ownership theory's expectation that these investors’ expanding asset holdings will predispose them to pressure their investees to address externalities (Fichtner and Heemskerk, 2020). For it illustrates that when these investors’ orientation towards maximizing the performance of markets is mediated by ESG investment's stipulation that investors should address only financially material risks, it often impedes financial markets’ internalization of public issues which do not pose a ‘systemic’ risk to the global economy. In the conclusion, we explore what implications these observations might hold for analyses of corporate governance under asset manager capitalism and reflect on the broader political implications of financial markets’ selective internalization of environmental and social issues.

## Conclusions

This article has examined the processes through which universal owners become concerned about the ethical, political and environmental issues surrounding meat and livestock production and through which some such issues begin to be internalized into financial markets. It has outlined a distinctly selective pattern of internalization in which issues perceived to present systemic risks to the future financial performance of firms across multiple sectors and industries, such as tropical deforestation, begin to materialize as matters of financial concern to universal owners. By contrast, the specificity of the issue of farm animal welfare to living animals which are found primarily within companies involved in intensive livestock farming has discouraged such investors from regarding it as a material risk to the overall performance of their portfolios. Those responsible for managing much of the capital invested in public equity markets, therefore, remain distinctly *un*concerned about farm animal welfare, hampering efforts to encourage institutional investor engagement with – and internalization of – this issue.

These observations support universal ownership theory's argument that universal owners’ economic interests are associated more closely with the aggregate performance of financial markets than that of individual assets (Braun, 2021; Hawley and Williams, 2000), and that they will therefore prioritize corporate governance interventions which mitigate negative externalities across entire markets over those which focus on individual firms (Fichtner and Heemskerk, 2020). However, they also highlight the need for refinement of these arguments by indicating that universal owners’ orientation towards maximizing aggregate economic performance shapes the ethico-political content as well as the manner of their stewardship of investee companies. For rather than predisposing universal owners to pressure their investee companies to internalize externalities in general, in practice this orientation towards maximizing the performance of markets over that of individual companies both stimulates *and* impedes financial markets’ internalization of public issues. Universal owners’ tendency to engage only with issues that present risks to the financial performance of their portfolio as a whole appears to *discourage* them from pressuring their investees to address concerns, such as farm animal welfare, which are material only to individual companies or sectors. Universal owners, therefore, pressure their investees to internalize negative externalities only very selectively – engaging with issues identified as presenting ‘systemic’ risks while permitting the continued externalization of those which impact only particular sectors or places.

As a result, the definitions, practices and calculative devices employed by universal owners to distinguish systemic risks from those of purely sectoral importance are assuming growing economic and political significance. These instruments of classification appear to play an increasingly prominent role both in defining the boundary between the financially immaterial and the material (and thus between the political and the economic) and in determining which issues may move across it. We have therefore argued that accounting for financial institutions’ selective engagement with, and financial markets’ selective internalization of, environmental and social issues requires that researchers not only analyse changing patterns of asset ownership but also attend to the ways in which changing technologies and practices of economic calculation reframe the boundaries of markets. Combining the theoretical tools of political economy and of marketization studies in this fashion has enabled this article to trace how ESG integration's assumption that investors should concern themselves only with issues that present material risks to their portfolios encourages universal owners to overlook sector-specific public issues and thus produces the uneven internalization of public issues outlined above.

This move is important because our analysis highlights that attempts to convince financial institutions to internalize new issues into their investment decisions and engagement programmes do not simply ‘politicize’ or ‘responsibilize’ finance. They also transform public issues through marketizing (or financializing) ethics and politics (Callon, 2009). Notably, in striving to concern universal owners about the environmental, social and health impacts of livestock production through demonstrating that these public issues will affect the future value of their asset portfolios, such initiatives often filter out certain aspects of the ethico-political disputes and programmes within which these issues originated. Concerns such as animal welfare, which are perceived as being of ‘merely’ ethical importance or as being material only to businesses within a single sector, fall away in the process of concerning mainstream financial institutions with meat and livestock issues. As a result, mainstream institutional investors’ efforts to internalize meat and livestock issues focus on a relatively narrow constellation of systemic risks, such as tropical deforestation, which implicate livestock agriculture but also affect varying constellations of other sectors.

This produces a peculiar phenomenon. The more fully a livestock issue becomes internalized into mainstream financial markets, the more divorced it tends to become from farm animals themselves. Issues such as tropical deforestation within food and feed supply chains become systemic risks which, although they play out through the health and appetites of animals, nonetheless overflow them. As investment in livestock production is reconceived as a vector of exposure to such systemic ESG risks, animals themselves are rendered oddly immaterial to deliberation about these issues. As such, efforts to internalize the politics of livestock into the operations of financial markets do not simply extend existing ethico-political debates into new sites of intervention and contestation but transform the content of public issues in ways that have yet to be fully explored. As the prevalence of ESG investment practices grows, financial markets are emerging as increasingly significant sites of problematization and negotiation for numerous environmental, social and ethical concerns. Examining the processes through which internalization into such markets reshapes public issues thus presents opportunities to understand what new erasures and externalizations these efforts to concern financial markets might occasion (and which old ones they reproduce) – and perhaps to contest the forms of neglect which they engender.
